# Unnecessary spine surgery: can we solve this ongoing conundrum?

**DOI:** 10.3389/fsurg.2023.1270975

**Published:** 2023-08-25

**Authors:** Khaled Fares AlAli

**Affiliations:** Zayed Military Hospital, Abu Dhabi, United Arab Emirates

**Keywords:** Spine, Spine surgery, Unnecessary surgery, UAE, neurosurgery, global surgery

“Do I need surgery?” This question has become increasingly common in my spine clinic over the past decade. After carefully examining the patient's medical history, performing a clinical exam and reviewing different radiographic imaging, I often find that surgery is not necessary in at least 80% of cases. This percentage is not derived from a scientific study, but it is consistent with the experiences of respected spine surgeons worldwide who have seen an overwhelming number of such cases in their clinics (1) and regional surgeons are not exempt from this issue (2–4).

Unnecessary surgery is defined as any surgical intervention that is either not needed, not indicated, or not in the patient's best interest when weighed against other available options, including conservative measures (5). In the spine, when the patients have no neurological deficit and no significant abnormal radiographic findings on dynamic x-rays, MR, and/or CT scans.

The first appearance of the term “unnecessary surgery” was when Dr. Paul Hawley, the Director of the American College of Surgeons, stated in an article in the New York Times (1953) “*the public would be shocked if it knew the amount of unnecessary surgery performed*” (5).

In a prospective observational study of 544 patients, 60% of the patients were recommended unnecessary spine surgery (USS) (6). This alarming statistic highlights the prevalence of USS and the need for a better understanding of its contributing factors and potential solutions. Several factors have been identified that contribute to the issue of USS. One of the primary etiologies is the overreliance on MRI findings. MRI is not a reliable tool for identifying the source of pain in patients with back or neck pain because degenerative findings are common in asymptomatic patients (7). This has been well established in the medical community since Jensen et al. published their findings in the New England Journal of Medicine in 1994 (8). They found that 52% of the patients without low back pain had disc bulge at least in one level, 27% had a disc protrusion, and 1% had a disc extrusion. Although international guidelines recommend against the use of imaging for routine diagnostic tests of lower back pain (9–11), several studies have identified an increase in imaging referrals for diagnostic imaging specifically MRI (12). Furthermore, wide spine practices variability due to lack of consensus in the management of the low back pain also plays a major role in increasing the practice of USS (9–11). Other causes of USS that have been described in the literature are wrong diagnosis, lack of knowledge of conservative management options, market competition, and financial gain (13). Stahel et al. (5) asked surgeons “why they need to do unnecessary surgery”. The two common answers were: USS were done because “we always have done this way” and for “financial gain, renown, or both”.

Moreover, USS can pose significant risks to patients without providing any benefits and can increase costs. For example, the estimated annual cost of USS is 4 billion in the United States of America (5), and the cost has increased significantly over the last two decades (6).

The issue of USS requires a multifaceted approach that incorporates various strategies and different measures. Second opinion programs in spine surgeries are helpful (14). It had been found to reduce at least 50% of the USS (6). However, there is a need to establish a clear second opinion guideline and define and recognize second opinion centers to achieve sound and unbiased reduction in USS.

Another way to reduce USS is a unified spine referral system. Spine surgery referrals through a multidisciplinary care pathway was found not only to reduce referral of nonsurgical spine cases but also increase referrals of patients who have clear surgical indications and reduce their waiting time (15). The American Board of Internal Medicine (ABIM) established on 2012 the Choosing Wisely campaign (CWC) which is a global initiative that aims to reduce unnecessary tests, treatments, and procedures in healthcare. The CWC can reduce unnecessary surgery and has active campaigns globally such as USA, Canada, Netherlands, Switzerland, Japan, Australia, the UK (16). and even regionally in Saudi Arabia (4). In 2014, North American Spine Society (NASS) joined the CWC and focus on elimination of unnecessary investigations for low back pain. According to a letter to its members, NASS was unable to make clear-cut recommendations for some common spinal procedures, such as spinal fusion due to insufficient evidence (17).

Governments have invested significant amount of money in improving the healthcare systems. It is our duty as healthcare providers to utilize all these resources to provide appropriate care to our patients. In my country, the United Arab Emirates (UAE), the USS is a growing concern (2, 3). Second opinion practices exist; however, I could not find any reference on its regulation. According to verbal discussions with some spine surgeons, insurance companies regulate second opinion program based on unknown criteria. The unnecessary surgeries issue is discussed between surgeons privately and not openly discussed in conferences or scientific meetings.

Therefore, I propose the following measures to the decision-makers to reduce USS and improve the quality of spine patient referrals. This measures apply to the UAE and several countries globally and regionally:

1. Setting up musculoskeletal clinics in primary healthcare centers to filter spine cases and prevent direct access to spine surgeons
2. Creating a multidisciplinary clinical pathway guideline for low back pain and implementing it in all musculoskeletal clinics in primary healthcare centers.
3. Implementing the second opinion referral to certain spine centers of excellence (preferably governmental) by insurance companies.
4. Creating a consensus expert opinion protocol and comprehensive practice guidelines for spine conditions in each country or region.
5. Establishing governmental centers of excellence for spine care affiliated with medical schools.
6. Limiting or monitoring the private sector's performance of spine surgeries.
7. Implementing medical ethics education and workshops for surgeons.
8. Creating a clear code of ethics for surgeons. Meanwhile, I encourage all surgeons and procedural medicine providers to read the statement of principles from the American College of Surgeons that includes pledge and code of professional conduct (18).
9. Including USS topic in the international spine conferences and allowing experts to discuss this issue openly.
10. Joining the CWC initiative by spine societies globally and regionally and revisit the new available evidence of some of the spinal procedures like fusion.

The issue of unnecessary spine surgeries has become a major concern globally and locally due to issues such as financial gain and resistance to change. Therefore, implementing proven measures such as musculoskeletal clinics and second opinion referral programs in spine centers of excellence can significantly reduce the incidence of unnecessary spine surgeries and improve patient outcomes. It is also essential for surgeons to prioritize the principles of professionalism and pledge to adhere to a code of professional conduct in their practice. Finally, it is important for medical professionals and decision-makers to openly discuss the issue of unnecessary surgeries, not only in spine surgery but also in other fields such as orthopedic surgery, plastic surgery, pain management procedures, interventional cardiology and the list goes on (5, 13)…
